# Genome Sequence of the Banana Plant Growth-Promoting Rhizobacterium *Bacillus amyloliquefaciens* BS006

**DOI:** 10.1128/genomeA.01391-15

**Published:** 2015-11-25

**Authors:** Rocío M. Gamez, Fernando Rodríguez, Johan F. Bernal, Richa Agarwala, David Landsman, Leonardo Mariño-Ramírez

**Affiliations:** a Corporación Colombiana de Investigación Agropecuaria (CORPOICA), Centro de Investigación Caribia, Magdalena, Colombia; b Corporación Colombiana de Investigación Agropecuaria (CORPOICA), Centro de Investigación Tibaitatá, Cundinamarca, Colombia; c National Center for Biotechnology Information, National Library of Medicine, National Institutes of Health, Bethesda, Maryland, USA; d BIOS Centro de Bioinformática y Biología Computacional, Manizales, Caldas, Colombia

## Abstract

*Bacillus amyloliquefaciens* is an important plant growth-promoting rhizobacterium (PGPR). We report the first whole-genome sequence of PGPR *Bacillus amyloliquefaciens* evaluated in Colombian banana plants. The genome sequences encode genes involved in plant growth and defense, including bacteriocins, ribosomally synthesized antibacterial peptides, in addition to genes that provide resistance to toxic compounds.

## GENOME ANNOUNCEMENT

Many root-colonizing *Bacillus* strains are beneficial for plant health and induce plant growth by mechanisms that include antagonizing soilborne pathogens. Banana root exudates containing organic acids and other compounds promote root colonization of plant growth-promoting rhizobacteria (PGPR) like *Bacillus amyloliquefaciens* (1). In the *B. amyloliquefaciens* genome, 9 giant gene clusters have been described that are dedicated to nonribosomal synthesis of antimicrobial compounds (2) and at least 46 genes are involved in carbon and nitrogen utilization in response to plant root exudates (3). *B. amyloliquefaciens* strains are known for promoting plant growth through diverse secondary metabolites (1) and are recognized as good candidates for use as biofertilizers in a variety of plants (4).

*Bacillus amyloliquefaciens* BS006 was isolated from *Physalis peruviana* (5, 6) roots in Boyacá, Colombia, and its PGPR properties were evaluated in tomato and banana plants. Genomic DNA was extracted from bacterial overnight cultures using the Ultraclean Microbial DNA kit MicroBead solution (MoBio, Carlsbad, CA, USA), modified with additional mechanical cell disruption using the MicroBead solution. Genomic DNA was prepared for Illumina sequencing using Agilent SureSelect QXT libraries. The whole-genome sequencing was performed using Illumina Hi ScanSQ, generating 10,785,126 single reads of 150 bp in length. The *B. amyloliquefaciens* BS006 genome was assembled using the reference-guided assembler ARGO, developed at NCBI, and the *de novo* assembler SPAdes (7), resulting in an assembly with a total of 4,173,094 bp, G+C content of 46.4%, and a genome coverage of 100.0×. Genome assemblies yielded 86 contigs with an *N*_50_ contig size of 287,634 bp. The contigs were annotated using the NCBI Prokaryotic Genomes Automatic Annotation Pipeline (PGAAP) and have been deposited at GenBank. The genome of *B. amyloliquefaciens* BS006 annotation revealed 4,128 genes, 3,998 coding sequences (CDSs), 6 rRNAs, 67 tRNAs, 1 noncoding RNA (ncRNA), and 56 pseudogenes.

The automatic annotation was enriched using RAST version 2.0 (8). We found in the genome of *B. amyloliquefaciens* BS006 a number of genes involved in the synthesis of 20 cofactors, 51 vitamins, and 20 pigments; 9 involved in potassium metabolism; 31 for nitrogen metabolism; and 31 for phosphorus metabolism. Additionally, the genome contains genes previously described for health and plant defense roles as follows: 1 adhesion, 16 bacteriocins and ribosomally synthesized antibacterial peptides, 37 resistance to antibiotics and toxic compounds, 17 invasion and intracellular resistance, and 117 dormancy and sporulation. All of these are genes that could be involved in plant growth-promotion and are probably related to the interactions between the rhizobacterium and its host *Musa acuminata* (banana Williams), suggesting diverse pathways potentially involved in banana plant growth. Here we announce the first genome sequence of the PGPR *Bacillus amyloliquefaciens* in Colombia.

### Nucleotide sequence accession numbers.

The draft genome sequence of *Bacillus amyloliquefaciens* BS006 has been deposited at GenBank under the accession number LJAU00000000. The version described in this paper is the first version, LJAU01000000. The BioProject accession is PRJNA236098.
